# List of Accidents Admitted into the Pennsylvania Hospital, from July 1st to July 30th, 1839

**Published:** 1839-08-10

**Authors:** Henry Wheaton Rivers

**Affiliations:** Resident Surgeon


### CLINICAL REPORTS.

### PHILADELPHIA HOSPITAL.

*List of Accidents admitted into the Pennsylvania Hospital, from July 1st to July 30th,* 1839.

[Reported by HENRY WHEATON RIVERS, M.D., Resident Surgeon.]

A case of compound fracture of the skull, caused by a bolt having a head three quarters of an inch in diameter, being driven through the frontal bone; it was forced in so firmly that his fellow-workmen were unable to extract it until surgical aid was called in; it is now five weeks since the accident and the patient is doing well.* A case of contusion, accompanied with some concussion of the spinal marrow and brain, caused by a fall from the fifth story of a house, treated by cups along the course of the spine and cold applications to head; removed from the house by his friends the day after the accident. A compound and comminuted fracture of the left foot and ankle, caused by a rail-road car passing over it; this case was admitted on the evening of the 4th of July, the leg was amputated immediately, the anterior tibial artery was found to be ossified, it was secured, however, by a ligature which has since come away; the patient, æt. sixty years, is doing well. A case of contusion of the hip, by a blow from a large log of wood, treated by the application of six wet cups to the part, and keeping at rest. A case of comminuted fracture of the left clavicle, accompanied with contusion of the right hip, caused by the passage of a loaded cart obliquely over the body; died in four days of mania a potu. A lacerated wound of the scalp, dressed with adhesive strips and cold wet cloths; died on the fifth day after his entrance of mania a potu. A case of lacerated wound of the scalp, caused by falling down a flight of stairs in an epileptic fit; the laceration was so extensive in this case that it was found necessary to bring the parts together with sutures, the patient had no bad symptoms and was discharged, cured twenty-four days after the accident. A case of lacerated wound of the scalp, including the right ear, which was partially torn off, produced by falling from a dray, the wheel of which passed over the side of the head. The treatment consisted in replacing the ear by means of sutures, a compress in the meatus, and a tight bandage round the head; the

* A full report of this case, which is a very interesting one, will be furnished at its termination.

wound is now healed. A case of punctured wound of the leg, from a stab with a large knife. A case of incised wound of the arm; died in four days of mania a potu. A case of compound and comminuted fracture of the left foot and ankle, from the passage of a rail-road car over it; this was a boy æt. five years; the leg was amputated by Dr. Norris, on the day of his entrance, (July 12th,) and the stump is doing well. A case of contusion of the ankle, caused by a blow from a ball in a ten-pin alley; treated by leeches, afterwards by soap-plaster, and keeping the leg at rest in a fracture-box; discharged cured in eleven days after the accident. A case of rupture of the ligament of the patella, produced by a fall on the knee while the leg was in a state of flexion; the treatment in this case was the same as would have been pursued in a fracture of the patella, viz., by elevating the limb on an inclined plane, a long splint on the under part of the leg, roller and compress. A case of contusion of the shoulder, caused by a blow upon the part, treated by cups, and rest; discharged cured thirteen days after the accident. A case of fracture of the clavicle near its outer end, treated by Dr. Fox's modification of Desault's apparatus. A case of contusion of the ankle, caused by being run over by a dray, treated by leeches, cold applications, and keeping the leg at rest in a fracture-box. A case of lacerated wound of the hand, from the bursting of a gun, treated by keeping the hand and arm at rest in a box of bran, with a view, if possible, of saving the hand; this patient was taken home by his friends two days after the accident. A case of contusion of the ankle, treated by soap-plaster, and being kept at rest in a fracture-box. A case of burn of both arms, caused by boiling water being spilt on them, dressed at first with olive oil and lime water, and afterwards with Goulard's cerate; doing well. A case of fracture of the ulna, about its middle, caused by a blow directly on the part, dressed in the usual way. A case of secondary hæmorrhage from a wound received three weeks before in the palm of the hand; a week before his entrance, he had hæmorrhage which was suppressed by a ligature round the radial artery, by Dr. G. M'Clellan; in this instance he applied to Dr. M'Clellan, who was absent from town, and on that account was sent to the hospital for the purpose of having the ulnar artery secured also, but there being no hæmorrhage when he arrived at the house, a compress was applied with a splint and tight bandage, cold applications were made use of, and the limb elevated on an inclined plane of pillows, since when, he has had no return of the hæmorrhage, and the wound is nearly healed. A case of wound of the hand caused by the discharge of a pistol, the wadding of which entered the palm of the hand, and was taken out five days after his entrance, (a poultice having been applied,) from the back of the hand near the thumb: a great deal of suppuration has taken place; the treatment has consisted in keeping the hand elevated and at rest; poultice to hand, and cold applications to arm. A case of contusion of the wrist from a blow directly applied, treated by keeping the arm and hand at rest in splints, and cold applications to wrist. A case of fracture of the neck of the thigh bone, in a female, æt. eighty-six years, treated by keeping the limb at rest on a double inclined plane of pillows. A case of compound and comminuted fracture of the leg, from the passage of a rail-road car over it; reaction did not take place until about two hours after his entrance; the leg was amputated on the following morning by Dr. Norris, and the stump is now doing well.

